# Ex vivo modelling of drug efficacy in a rare metastatic urachal carcinoma

**DOI:** 10.1186/s12885-020-07092-w

**Published:** 2020-06-23

**Authors:** Rami Mäkelä, Antti Arjonen, Ville Härmä, Nina Rintanen, Lauri Paasonen, Tobias Paprotka, Kerstin Rönsch, Teijo Kuopio, Juha Kononen, Juha K. Rantala

**Affiliations:** 1 Misvik Biology Ltd, Karjakatu 35 B, FI-20520 Turku, Finland; 2 Brinter Ltd, Turku, Finland; 3 University of Sheffield, Sheffield, UK; 4 Central Finland Health Care District, Jyväskylä, Finland; 5 UPM-Kymmene Corporation, Helsinki, Finland; 6 Eurofins Genomics Europe Sequencing GmbH, Constance, Germany; 7 Docrates Hospital, Helsinki, Finland

**Keywords:** Ex vivo drug screening, Urachal carcinoma, Rare cancer, Precision medicine

## Abstract

**Background:**

Ex vivo drug screening refers to the out-of-body assessment of drug efficacy in patient derived vital tumor cells. The purpose of these methods is to enable functional testing of patient specific efficacy of anti-cancer therapeutics and personalized treatment strategies. Such approaches could prove powerful especially in context of rare cancers for which demonstration of novel therapies is difficult due to the low numbers of patients. Here, we report comparison of different ex vivo drug screening methods in a metastatic urachal adenocarcinoma, a rare and aggressive non-urothelial bladder malignancy that arises from the remnant embryologic urachus in adults.

**Methods:**

To compare the feasibility and results obtained with alternative ex vivo drug screening techniques, we used three different approaches; enzymatic cell viability assay of 2D cell cultures and image-based cytometry of 2D and 3D cell cultures in parallel. Vital tumor cells isolated from a biopsy obtained in context of a surgical debulking procedure were used for screening of 1160 drugs with the aim to evaluate patterns of efficacy in the urachal cancer cells.

**Results:**

Dose response data from the enzymatic cell viability assay and the image-based assay of 2D cell cultures showed the best consistency. With 3D cell culture conditions, the proliferation rate of the tumor cells was slower and potency of several drugs was reduced even following growth rate normalization of the responses. MEK, mTOR, and MET inhibitors were identified as the most cytotoxic targeted drugs. Secondary validation analyses confirmed the efficacy of these drugs also with the new human urachal adenocarcinoma cell line (MISB18) established from the patient’s tumor.

**Conclusions:**

All the tested ex vivo drug screening methods captured the patient’s tumor cells’ sensitivity to drugs that could be associated with the oncogenic KRAS^G12V^ mutation found in the patient’s tumor cells. Specific drug classes however resulted in differential dose response profiles dependent on the used cell culture method indicating that the choice of assay could bias results from ex vivo drug screening assays for selected drug classes.

## Background

The development of high-throughput screening technologies and cell culture methods has made it feasible to perform large-scale in vitro drug screens also using patient derived primary tumor cell cultures [1–3]. These techniques are collectively called as ex vivo drug screening methods. The utility of ex vivo drug screening has emerged as a novel approach to complement pathological cancer diagnostic procedures to track patient specific drug sensitivity to hundreds of cancer therapeutics in a single experiment [3]. The results can be used to confirm drug sensitivity patterns predicted from molecular genetics [2] or to inform treatment decision and personalized care of individual cancer patients when standard treatment options have been exhausted [3]. In context of rare cancers, the low number of patients limits the clinical evaluation and validation of novel treatment strategies using conventional trial mechanisms. Thus, demonstration of the efficacy of novel therapeutics in rare cancer types through empirical evidence from ex vivo tests or similar alternative models may be the only option to motivate clinical development of these treatments [3–5]. One such rare cancer, for which ex vivo evidence could be used as motivation for development of novel treatment strategies, is urachal cancer, an aggressive non-urothelial bladder malignancy accounting for less than 1% of all bladder cancers [6]. Urachal adenocarcinoma (UrAC) arises in adults from the vestigial musculofibrous remnant band that connects the allantois and the bladder during embryonic development. A large proportion of patients with UrAC initially present with an advanced disease [7] and patients with metastatic urachal cancer have a poor prognosis [8–11]. Given the rarity of urachal cancers, prospective trials to guide the treatment of patients with advanced disease are lacking, there are no standard chemotherapeutic regimens, and surgery remains the mainstay of therapy shown to improve the overall survival outcome of UrAC [12]. To date, no randomized trials of urachal carcinomas have been reported and the most comprehensive reviews to date have concluded 420 [10] and 456 [11] patients reported regionally and 1010 patients reported globally [13]. As a result, limited information exists regarding the effective management of these cancers beyond the use of chemotherapy including 5-fluorouracil based, 5-fluorouracil and cisplatin [14–17] or hyperthermic intraperitoneal chemotherapy [18–20]. Especially, knowledge concerning the efficacy of new genome aberration targeted chemotherapeutic agents is limited to a handful of case reports from individual institutions [14, 21–25]. Moreover, comprehensive tumor genomic profiling of UrAC samples has not been described and the only common genetic features described in the limited number of reported cases have included aberration of APC, BRAF, EGFR, KRAS, PIK3CA, TP53 and microsatellite instability [21–26]. To improve our understanding of the disease pathogenesis and therapy sensitivity of UrAC, we performed a large-scale ex vivo drug screening of 1160 drugs with vital tumor cells derived from a patient with a metastatic urachal adenocarcinoma. In this study, we also compared the reproducibility of results derived with three different high-throughput drug screening approaches to assess assay dependency of the ex vivo measured dose responses of the patient derived tumor cell cultures. Last we describe establishment of a new human urachal adenocarcinoma cell line (MISB18) which is the first described UrAC cell line with a known genetic background.

## Methods

### Patient derived primary tumor cell culture

**Fig. 1:**
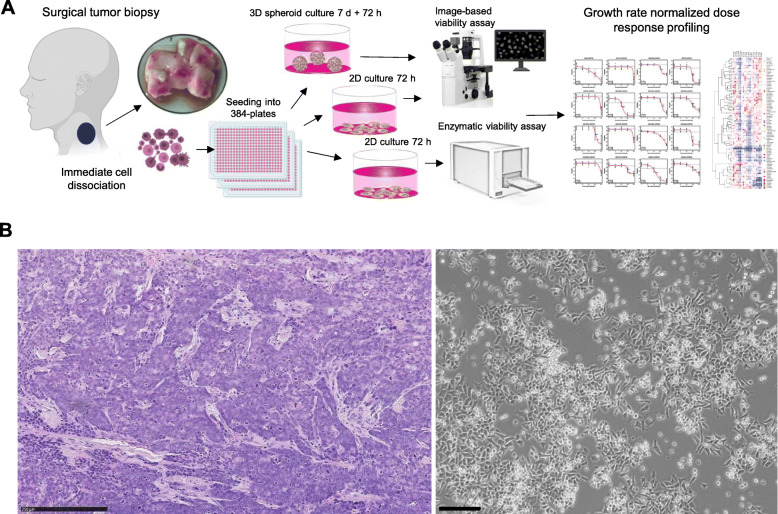
Overview of the study design and the patient sample. **a** A surgically resected tumor sample from a metastatic nodule on the patient’s neck was received for the study. Tumor cells were isolated on day of surgery and used immediately for ex vivo drug screening of 1160 drugs. 2D and 3D cell culture assay approaches were used in parallel to evaluate reproducibility of the results. Image-based assays and the enzymatic cell viability assay results were normalized using growth rate normalization. **b** Left: Haematoxylin & eosin staining of the metastatic urachal adenocarcinoma tissue showing poorly differentiated neoplastic cells, bar 250 μm. Right; transmitted light microscopy image of the tumor derived cell culture at day 4 after dissociation of the cells, bar 100 μm

The patient was identified to the study by an oncologist at Jyväskylä Medical Central, Finland. A subcutaneous metastasis tissue sample was collected for the study during palliative surgery. In conjunction with the surgical procedure, part of the dissected tumor tissue was placed in sterile RMPI-1640 medium (Gibco) for transport to the consulting pathologist for preparation and further delivery to the research laboratory (Fig. 1a). Rest of the tissue was fixed in 4% buffered formaldehyde, paraffin-embedded, cut at 4 μm, and subjected to routine staining procedures including hematoxylin and eosin stain (H&E) and pathological evaluation (Fig. 1b). The live tissue was dissociated into a single cell suspension as described before [3]. Following the enzymatic dissociation, the resulting cell suspension was counted using a Cellometer Mini cell counter (Nexcelom). In total 6.5 × 10^6 cells with an average size of > 13 μm was derived from ~2cm^3^ of the tumor tissue. The suspension was diluted to RPMI-1640 medium (Gibco) containing 5% FBS to achieve a suspension with 1000 cells per 45 μL of medium. 5 × 10^6 cells were used for the initial ex vivo drug screening and the rest were placed to cell culture in standard cell culture conditions (37 °C, 5% CO_2_). Following 4 days in culture, the cells presented a semi-adherent phenotype with cells growing both as loose aggregates and adhered to the plastic cell culture surface (Fig. 1b). The use and investigation of the patient derived cells was approved by the local Ethics Committee of the Central Finland Health Care District (KSSHP 3 U/2015). All the experiments were undertaken with the understanding and written informed consent of the patient. The study methodologies conformed to the standards set by the Declaration of Helsinki.

### Ex vivo drug screening

The therapeutic compound collection used in the ex vivo study consisted of 1140 FDA approved drugs, purchased as single a collection of FDA approved drugs from a commercial chemical vendor (Cat.no. L1300, Selleck biochemicals, Houston, TX, USA) readily dissolved in dimethyl sulfoxide (DMSO). The library was supplement with 20 investigational and preclinical compounds covering key cancer associated signaling pathway targets including AKT, ATR, BET (bromodomain), EGFR, FGFR, MDM2, MEK, PIM, PI3K, pan-RAF and WEE1. Since platinum-based drugs (cisplatin, carboplatin and oxaliplatin) are inactivated by DMSO, these were replaced in the compound library with stock solutions diluted in physiological saline. The ex vivo drug screening experiments were performed in 384-well microplate format as described before [3]. Briefly, each compound was tested in the initial high content image-based screening with three different concentrations in 2-fold dilutions starting from 5 μM as the highest concentration. In the secondary screening experiments, the compounds were tested in five 2-fold concentrations starting from 5 μM as the highest concentration. For the screening experiments performed with 2D cell cultures, the compounds were pre-printed on tissue culture treated 384-well plates (Corning, ThermoFisher Scientific) diluted in 5 μl of RPMI-1640 medium without supplements with a liquid handling device (Eppendorf EpMotion-96, Eppendorf GmbH.). For the screening experiment performed with 3D cell cultures, the compounds were aliquoted on top of the 3D cell cultures in 10× concentration. Cell suspension of freshly isolated urachal carcinoma cells (45 μl per well; 1000 cells per well) was then transferred to each well using Multi-Drop Micro peristaltic dispenser (ThermoScientific). The 384-well plates were then incubated for 72 h at 37 °C and 5% CO_2_.

### Enzymatic cell viability assay

To assess drug induced growth inhibition with an enzymatic cell viability assay following a 72-h incubation of the cells with drugs, cell viability was measured using CellTiter-Glo reagent (Promega) according to the manufacturer’s instructions with Labrox luminescence plate reader (Labrox). Briefly, 20 μL of the reagent was added per 384-well and incubated for 30 min at room temperature in gentle shaking. Cell viability luminescence data was normalized to median luminescence signal from 0.05% DMSO only wells (negative controls), 2 mM hydroxyurea containing wells (proliferation growth controls) and 5 μM staurosporin containing wells (positive controls). Dose response was presented as growth rate (GR) normalized % of signal in comparison to negative and positive control samples (see Statistical analysis).

### Image-based cell viability assays

Microscopic image-based drug screening with 2D cell cultures was performed using an Olympus scan^R integrated high content imager and image analysis suite (Olympus) equipped with a Hamamatsu ORCA-R2 CCD digital camera (Hamamatsu Photonics K.K). Each well was imaged individually with a 10× objective using specific filter sets for DAPI (Semrock). The scan^R image analysis software suite was used for quantitative analysis of image features. Analysis capabilities included cell segmentation based on nuclear DNA staining and cell counting. The effects of each drug on cell counts as indicator of cell growth inhibition or cytotoxicity were assessed by comparing cell counts with comparable cell counts measured in DMSO only wells (negative controls) and 2 mM hydroxyurea containing wells (proliferation controls). The DNA counterstaining of the cells was performed according to the following protocol. First the culture medium was aspirated carefully from each well and the cells were fixed with 2% paraformaldehyde (Sigma-Aldrich) in PBS for 15 min at room temperature. Cells were then washed once for 5 min with PBS. Cells were permeabilized with 0.3% Triton-X100 in 20 μL of PBS for 15 min at room temperature, followed with a PBS wash. DAPI (4′,6-diamidino-2-phenylindole, Invitrogen) DNA counterstaining was performed for 1 h at room temperature, followed by washing with PBS (Supplementary Figure 1).

### 3D cell culture assays with imaging cytometry

The 3D cell culture assays were performed using GrowDex® hydrogel (UPM, Helsinki, Finland) as the matrix supporting 3-dimensional cell growth. To minimize adherent cell growth in the bottom surface of the wells, the microwells were first coated with 1.2% pHEMA (poly (2-hydroxyethyl methacrylate; Polysciences). For the experiment the 1.5% hydrogel stock was mixed with complete cell culture medium (RPMI-1640 + 5% FBS) to achieve a 0.6% w/v hydrogel solution. 30 μL of cell suspension containing 2000 cells was mixed with 30 μL of the diluted hydrogel to achieve a 0.3% w/v hydrogel solution containing ~ 30 cells/μL. Required amount of hydrogel-cell solution was prepared by pipetting the hydrogel using a wide-mouth 10 mL pipetting tip into a 50 mL tube and gentle mixing with a vortex shaker. The hydrogel-cell solution was dispensed to 384-plate wells using a Multidrop plate dispenser (ThermoFisher Scientific). The total volume of sample added into a single 384-well was 60uL/well (2000 cells). Following dispensing, plates were centrifuged for 1 min at 100 g and left on incubation at + 37 °C, 5% CO_2_ for 7 days prior to addition of the drugs. At day 4, 20 μL of medium was carefully aspirated from the wells using a multichannel pipet and 20 μL of fresh complete cell culture medium was added to each well. On day 7, the same was repeated followed by addition of 6 μL of the 10× drug stocks per well and additional 72-h incubation. For analysis of cell growth, the cells were stained using Hoechst 33342 cell permeant live cell DNA dye (Invitrogen). 8 μL of 10× stock dilution was added per well and incubated for 45 min. Following Hoechst staining, the plates were centrifuged for 2 min at 200 g to settle the cell spheroids/aggregates to the bottom plane of the wells (Supplementary Figure 1). Imaging and image analysis were then performed using an Olympus scan^R high content imager and image analysis suite as described above for the 2D assays.

### Mutation analysis

Targeted genomic profiling of an oncopanel with 850 cancer associated genes was performed from the ex vivo tumor cell culture following 1 month in culture. Genomic DNA was extracted from 1 × 10^6 cells using NucleoSpin Tissue (Macherey-Nagel GmbH) DNA purification kit according to the manufacturer’s protocol. Hybridization-based target capture was performed with Agilent SureSelect (Agilent) technology and sequencing libraries were sequenced using paired end 100 bp read format on an Illumina HiSeq2500 instrument per the manufacturer’s instructions (Illumina). Single nucleotide variants (SNVs), insertions and deletions (In/Del) were detected and filtered based on mutation allele frequency (> 1%). Detected variants were screened for known clinical significance in ClinVar (released 02. Oct 2017) database [27]. Result are available online at Mendeley data; DOI: 10.17632/kc7wmn3rcs.2.

### Statistical analysis

Statistical analysis was performed with the Microsoft Excel, Cluster 3.0 and GraphPad Prism 7 statistical software. The ex vivo drug screening data was analyzed using the normalized growth rate inhibition (GR) approach which yields per-division metrics for drug potency and efficacy. The normalized growth rate inhibition (GR) method corrects for variation in division rates by estimating the magnitude of drug response on a per cell-division basis. The GR values were used for comparison of drug potency between the different screening methods to correct for differential proliferation rate of the cells in 2D and 3D culture conditions. GR values were calculated as previously described [2, 3]. Combination indices (CI) were calculated from replicate, fixed-ratio, dose escalation experiments using the Chou and Talalay method [28]. CI values were reported at 50% inhibitory values (CI50). IC50 values were calculated with GraphPad Prism 7 software using a nonlinear curve fit equation. Due to the limited number of primary cells available for technical replicate screening experiments, different drug test doses were considered as biological replicates, and the corresponding *p*-values were calculated across the dilution series with Welch’s t-test according to assumptions on data normality.

## Results

### Ex vivo drug screening of urachal cancer cells

**Fig. 2:**
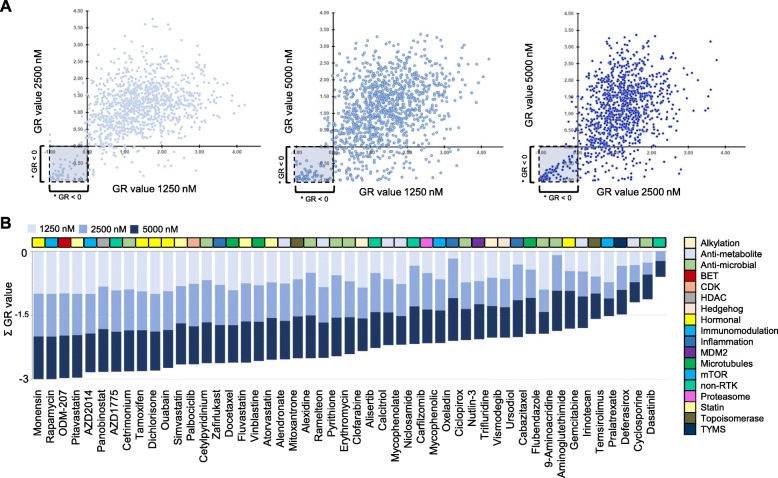
Large-scale ex vivo drug screening in patient derived urachal carcinoma cells. **a** Scatter plots comparing the GR value correlation of all the drugs in the different test concentrations. A compound library of 1160 drugs was used to assess cytotoxicity on urachal cancer cells following 72-h exposure. Analysis was performed using an imaging cytometry assay and GR scoring. Drugs reducing cell viability more that the proliferation stalling control hydroxyurea (GR < 0) in all concentrations were considered significant. **b** 49 most effective cytotoxic compounds reducing cell viability (GR < 0) across all test concentrations. GR values of the 3 drug doses stacked and compounds ordered by the averaged cytotoxicity. Drug target/class of each drug is indicated with the different colors

The patient, a 36-year-old male was diagnosed with a 9 cm cystic-solid tumor in front of bladder. The tumor cells infiltrating bladder epithelium demarcated sharply from the urothelium. Initial treatment included cystectomy and wide pelvic lymphadenectomy. Pathological evaluation of the surgical preparation confirmed the diagnosis of urachal mucinous cystadenoma and poorly differentiated urachal carcinoma. Carcinoma fraction of the tumor contained various different regions with mixed histological features, including partial differentiation to urothelial carcinoma, intestinal carcinoma and squamous cell carcinoma. Cancer showed high proliferation rate, mitotic figure count was 40/10 HPF. Up to 30% of the tumor contained necrotic tissue. Adjuvant treatment of the patient consisted of six cycles of cisplatin – 5-FU regimen. Immediately after adjuvant chemotherapy patient presented with subcutaneous metastatic lesion located behind left ear. Patient received radiotherapy with a palliative intent (30 Gy, 10 × 3 Gy). Disease progressed shortly after completing radiotherapy course and second-line systemic chemotherapy was initiated with docetaxel – gemcitabine. Disease progressed again and new lesions appeared in multiple locations (neck, axilla, sternum, adrenal gland, peritoneal region, sacrum). At this stage, palliative debulking surgery was performed on a painful subcutaneous lesion on the neck [29]. A section of tumor tissue was collected for the purpose of ex vivo therapy efficacy screening and a section was prepared for histopathology confirming metastatic urachal adenocarcinoma showing poorly differentiated neoplastic cells (Fig. 1b). Using standard techniques to establish cell cultures from human tissues [30], a primary cell culture was prepared for the drug screening experiment on the day of the surgery. Cytotoxicity of 1160 drug compounds representing all different FDA approved drug classes, with a fixed dose range of 1.25 μM to 5 μM was performed with the primary tumor cell culture (Mendeley data: DOI: 10.17632/kc7wmn3rcs.2). With comparison of the negative control samples and hydroxyurea treated proliferation control samples, the estimated cell doubling rate of the primary culture was calculated to be 240 h corresponding to ~ 0.3 cell division over the course of the 72-h assay. To identify the most potent growth inhibitory drugs, effects of the drugs were averaged across all the test concentrations to derive a stringent ranking criterion where the growth inhibitory impact of the drug had to be stronger than the cell growth stalling effect of hydroxyurea (GR < 0) across all test doses (Fig. 2a). From this, 49 drugs resulting in a strong cytotoxic effect were nominated (Fig. 2b). These included 19 drugs used or developed for anticancer purposes (antimetabolites, microtubule poisons, nucleoside analogs, topoisomerase inhibitors, targeted therapeutics including WEE1, CDK4/6, mTOR, HDAC and a novel BET inhibitor ODM-207), as well as 30 drugs with other indications including statins and antibiotic compounds (Fig. 2b). The most potent growth inhibitory compound from the panel of 1160 drugs was Monensin, a monocarboxylic acid ionophore veterinary drug produced by *Streptomyces cinnamonensis* with antibiotic and anticancer activity [31, 32].

### Validation using different ex vivo screening techniques

**Fig. 3:**
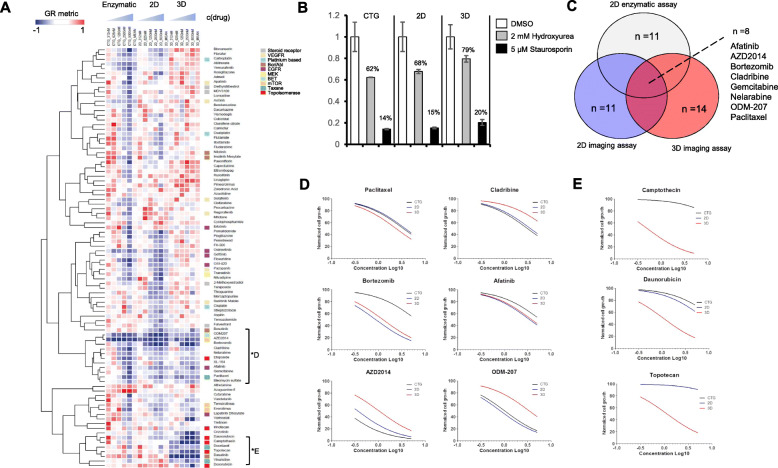
Ex vivo validation screening using different assay techniques. **a** Heatmap display of vertical unsupervised clustering of the dose response data of the drugs from independent ex vivo screens using a 2D enzymatic cell viability assay, an image-based 2D cell viability assay and an image-based 3D cell viability assay. Each drug was tested in five concentrations. GR values < 0 shown in blue. **b** Comparison of the assay controls used to calculate growth rates of the patient derived cells in the 2D and 3D culture conditions and measured with the different assay techniques. **c** Venn diagram showing the overlap of the top 30 most cytotoxic drugs from the different assay techniques. **d** Curve fitted dose response curves of the most potent cytotoxic drugs across all three different replicate screens. **e** Topoisomerase inhibitors had systematically a more potent cytotoxic effect in the 3D cell culture model assay

To validate findings from the primary drug screen and to evaluate dependency of the drug efficacy profiles on the used assay technique, a repeated analysis of 90 selected drugs with an expanded dose range was performed using three different high-throughput screening approaches; an enzymatic 2D cell viability assay, an imaging-based 2D cell viability assay and an image-based cell viability assay of cells cultured in 3D cell culture conditions (Fig. 3a). All drugs were tested with five 2-fold concentrations and dose responses were normalized using GR metrics to correct for the differential measured cell growth rate of ~ 0.6, ~ 0.5 and ~ 0.3 cell doublings per 72 h in the image-based 2D, enzymatic 2D and image-based 3D assay respectively (Fig. 3b). Unsupervised hierarchical clustering of the dose responses across all three different assay methods was performed to identify patterns of response among the drug classes and to visualize variation in the response dependent on the used assay method (Fig. 3a). The overall most cytotoxic drugs independent of the assay method, based on growth rate normalized dose responses, were afatinib (2nd generation EGFRi, IC50 3,83 μM), AZD2014 (mTOR1/2i, IC50 0,36 μM), bortezomib (proteasome inhibitor, IC50 1,24 μM), cladribine (purine analogue, IC50 3,57 μM), ODM-207 (bromodomain inhibitor, IC50 1,01 μM) and paclitaxel (taxane, IC50 3,38 μM) (Fig. 3c, d). The median IC50 of mTOR inhibitor AZD2014 (vistusertib) when measured across the three different assay methods was lower by ~ 10× to ~ 20× in comparison to 5-FU, docetaxel, ciplatin or gemcitabine, agents that had been used to treat the patient. Interestingly, all included topoisomerase inhibitors; camptothecin, doxorubicin, irinotecan and topotecan resulted in more potent cytotoxic effects in the 3D cell culture assay (Fig. 3e) [33]. Overall, the drug response data measured with the enzymatic 2D assay and the image-based 2D assay had the highest degree of concordance across the full dose range (Pearson correlation, average across all doses, *r* = 0.45). In general, the cytotoxic effects of majority of drugs were less potent in the 3D assay with the exception of topoisomerase inhibitors, docetaxel and vincristine.

### Evaluation of targeted drug combinations

Results of the ex vivo screening indicated an apparent MAPK/PI3K signaling pathway switch in the tumor cells based on the high sensitivity of the cells to mTOR inhibition, MEK inhibition and partial response also to antifolate abitrexate, BRAF and EGFR inhibition. Other targeted therapeutics displaying cytotoxic effects were the ALK/ROS1/cMET inhibitor crizotinib, experimental bromodomain inhibitor ODM-207 and several VEGFR angiogenesis inhibitors (Fig. 3a). Efficacy of these compounds varied only little between the different ex vivo assay techniques indicating no dependency on the mode of cell growth (2D vs. 3D) (Fig. 3a). The RAS/RAF/MEK/ERK and PI3K/AKT/mTOR signaling pathways all belong to mitogen-activated protein kinase (MAPK) signaling pathways. Mutations and/or activation by other mechanism of any one of the upstream genes (such as KRAS, BRAF, EGFR or MET) may result in abnormal activation of the signaling pathway converting into sensitivity towards inhibition of the MAPK signaling pathway [34]. Comparison of the sensitivity of patient derived tumor cells with the sensitivity of primary cultures previously derived from three urothelial bladder carcinomas and one small-cell neuroendocrine bladder carcinoma with no known MAPK pathway activating mutations [1] confirmed the selective sensitivity of the urachal cancer cells to the mTORC1/2 inhibitor AZD2014, MEK inhibitor trametinib and EFGR inhibitor afatinib (Supplementary Figure 2). To assess the genetic background of the patient’s tumor cells a targeted oncopanel DNA sequencing was performed from tumor cells kept in continuous culture for 1 month following the drug screening experiments. Two known pathogenic mutations associated also with urachal cancers; FGFR4 and KRAS [23] and seven known mutations associated with a drug response were identified in the patient’s tumor cells (Table 1.).

**Table 1:** Clinical significance SNVs identified in the patient’s tumor cells

Gene	AA Change	Codon Change	Mutation freq.	ClinVar ID	ClinVar significance
ABCB1	p.S829A, pS893A	c.2677 T > G, c.2485 T > G	31,8%	rs166622	drug response
DPYD	p.M166V	c.495A > G	47,1%	rs100116	drug response
FGFR4	p.G23R, p.G388R	c.67G > A, p.1162G > A	100,0%	rs16326	pathogenic
KRAS	p.G12V	c.35G > T	71,6%	rs12583	pathogenic
SLCO1B1	p.V174A	c.521 T > C	99,8%	rs37346	drug response
TAS2R38	p.I296V	c.886A > G	99,9%	rs2906	drug response
TAS2R38	p.A49P	c.145G > C	100,0%	rs2904	drug response
TP53	p.P33R, p.P72R	c.98C > G, c.215C > G	99,7%	rs12351	drug response
XPC	p.Q939K	c.2815C > A	100,0%	rs190215	drug response

**Fig. 4:**
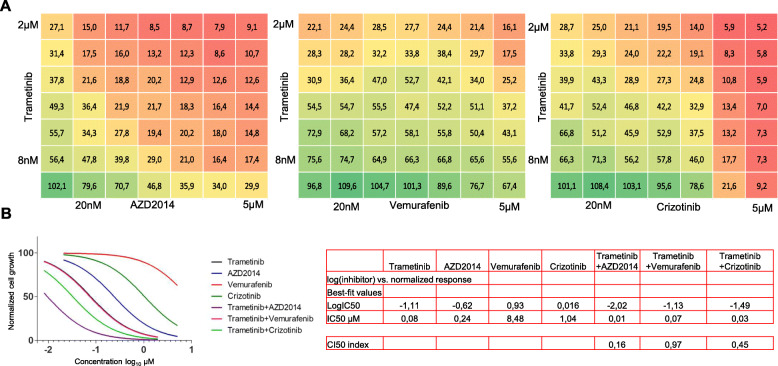
Evaluation of potent drug combination strategies in urachal cancer cells. **a** Dose–response matrices of percent of cell viability resulting from exposure to the indicated drug combinations. Drugs were tested in 6 concentration in a matrix covering all possible combination of the dilutions in triplicate. Cells were exposed to the drugs for 72 h in 2D cell culture. **b** Curve fitted dose response curves and a table with the IC50 estimates of the single agents and the CI50 combination index values for the drugs at a fixed molar ratio of 1 to 2.5

As suggested by the responsiveness of the patient’s cells to MEK inhibitor trametinib [34], the tumor cells were found to harbor a somatic activating KRAS^G12V^ mutation reflecting results from earlier studies reporting KRAS mutations being more common in UrAC than in urothelial bladder cancers [12]. For an in-depth view on potential drug combinations that could be synergistic with MEK inhibition in the treatment of KRAS mutated UrAC, we explored the effects of combining MEK inhibitor trametinib with the mTORC1/2 inhibitor AZD2014, ALK/cMET inhibitor crizotinib and BRAF inhibitor vemurafenib having cytotoxic effects both on the 2D and 3D assays (Supplementary Figure 1). The MISB18 cell line established from the patient’s tumors were tested with a matrix of the inhibitor combinations in six doses. Trametinib was administered in six 3-fold dilutions starting from 2 μM and the other drugs in six 3-fold dilutions starting from 5 μM. Combination treatments with MEK and mTOR inhibition and MEK and ALK/cMET inhibition revealed additive effects of cytotoxicity (CI50 = 0.16, CI50 = 0.45 respectively), resulting in significant net reduction in cell numbers following 72 h of treatment (Fig. 4a). Cytotoxic IC50 of trametinib and AZD2014 as a combination at a fixed molar ratio of 1 to 2.5 was < 10 nM, compared to IC50 values of 80 nM and 240 nM of the drugs as single agents, respectively (Fig. 4a & b). This finding is consistent with the role of kinome reprogramming and the alternate RTK signaling pathways feeding in the development of acute resistance to MEK inhibition cancer cells [35]. Also, the synergistic effect of inhibition of ALK/ROS1/cMET upstream of the MAPK pathway in combination with MEK inhibition fits concept as ROS1 inhibition has been previously shown to potentiate the anticancer effect of trametinib [35].

## Discussion

The last decade of cancer research marks the era for evolution of the concept of personalized cancer care through the revolution of genetics and targeted therapies. In clinical oncology settings, the application of genomic medicine has been pioneered towards clinical trials where systemic cancer treatment is being targeted to individual patients based on molecular characterization of the patient’s tumor [36]. This is particularly promising in context of clinical care of rare cancers, for which large clinical studies are not possible due to low number of cases. Treatment of rare cancers is often based on empirical approaches with standard chemotherapies. With advanced disease these treatments often fail, and no additional therapeutic options are available due to lack of clinical evidence on targeted treatments. In the future, combination of diagnostic therapy efficacy screening with genomic information [1–3, 37–40] could provide a robust diagnostic approach for personalized cancer medicine including immuno-oncology therapies [41] and thereby shift the clinical practice paradigm also in rare cancers. Currently the ex vivo screening techniques are under intense development and multiple different approaches has been described by different research groups [1–3, 37–41]. A common topic of debate regarding the ex vivo drug screening methods is the in vivo representativeness of the used models. 3D cell culture models have been promoted to increase the success rate of primary tumor cell cultures and resemble the primary tumors better than traditional two-dimensional (2D) cell culture models. This is in part due to potential transformation and loss of heterogeneity of cancer cell cultures under conventional 2D in vitro propagation. Here, both 2D and 3D cell culture conditions were tested in parallel to compare the reproducibility of the methods. While the image-based and enzymatic 2D cell culture assays had the best overall correlation of the dose response results, all three techniques yielded dose and target dependent cytotoxic profiles for drugs which could be linked directly to genomic features of the patient’s cancer. Many drugs had also a significantly stronger cytotoxic effect in both the 2D and 3D assay than the standard chemotherapeutics that are being used for treatment of UrAC including the current patient. This shows that ex vivo therapy efficacy screening could be used as a rapid technique to complement pathological and clinical diagnostics to inform on treatment decisions [3]. Indeed, results from the first reported clinical trial utilizing ex vivo drug screening indicated a 88% overall response rate (ORR) for patients treated on basis of the approach [37]. This suggest that ex vivo screening has the potential for high accuracy in predicting responsive patients.

## Conclusion

To evaluate feasibility and reproducibility of different ex vivo drug screening approaches to model therapeutic options for metastatic urachal adenocarcinoma, we performed a large-scale ex vivo drug screening using tumor cells freshly isolated from an UrAC tumor biopsy. The primary drug screening of 1160 drugs was initiated on the day of surgery and the screening results were available 4 days after sampling of the tissue. Findings from the initial screening were confirmed by alternative ex vivo screening techniques based on 2D and 3D cell culture models and two different assay approaches. All different assay techniques suggested sensitivity of the patient’s tumor cells towards inhibition of MAPK signaling pathway targets MEK and mTOR. Targeted NGS profiling of the patient’s cells confirmed an activating KRAS^G12V^ mutation giving a rationale for the increased sensitivity of the tumor cells to MAPK signaling pathway. While our study is limited by the analysis of only a single UrAC patient sample, the significance of the results is the demonstration that rapid ex vivo screening without prior in vitro propagation of the patient derived tumor cells, both with 2D and 3D cell culture systems, identified drug sensitivities that reflected the genomic profile of the patient’s tumor. Moreover, we describe the first UrAC cell line (MISB18) with a known tumor genomic profile, which together with future analyses of additional ex vivo UrAC samples can be used as a model to establish the role for pathogenic KRAS mutations on UrAC pathophysiology and drug sensitivity.

## Supplementary information

**Additional file 1: Figure S1.** Microscopic imaging of 2D and 3D urachal cancer cell cultures. Example transmitted light microscopy images of the phenotypes for the cytotoxic drugs at 1250 nM concentration identified as potential therapeutics for urachal cancer cells based on the 2D and 3D ex vivo drug screening. Both brightfield microscopy and fluorescence microscopy imaging with DNA counterstaining with Hoechst (3D cultures) and DAPI (2D cultures) was performed with a 10× objective on an Olympus scan^R high content imager, bars 100 μm.

**Additional file 2: Figure S2.** Comparison of the drug primary urachal cancer cell culture to patient derived bladder cancer cell cultures. A) Heatmap visualization of the dose response of the urachal cancer cells MISB18 with four cell cultures established from patients samples of different bladder cancer types. GR values of < 0 shown in blue. B) GR metrics describing the sensitivity of the cells to three drugs; afatinib, AZD2014 and trametinib displaying strongest selective cytotoxic effects on the urachal cancer cells in comparison to the bladder cancer cell cultures (data from image-based screening assays). Data available at Mendeley Data; DOI: https://doi.org/10.17632/kc7wmn3rcs.2.

## Data Availability

All data generated or analyzed during this study are included in this published article [and its supplementary information files]. Supplementary data is deposited to Mendeley data; DOI: 10.17632/kc7wmn3rcs.2. The patient derived urachal cancer cell line MISB18 has been kept in continuous culture for an excess of 80 passages and is available from the corresponding author on reasonable request for non-profit research purposes in accordance to a specified MTA.

## Abbreviations

2D: 2 dimensional
3D: 3 dimensional
ALK: Anaplastic lymphoma kinase
EGFR: Epidermal growth factor receptor
FGFR4: Fibroblast growth factor receptor 4
KRAS: KRAS proto-oncogene
mTOR: mammalian target of rapamycin
MAPK/MEK: Mitogen-activated protein kinase
NGS: Next-generation sequencing
UrAC: Urachal adenocarcinoma
