# Supplementary figures and images for: Ex vivo modelling of drug efficacy in a rare metastatic urachal carcinoma
Source: BMC Cancer. 2020 Jun 23;20:590. doi: 10.1186/s12885-020-07092-w (PMC7313172; doi:10.1186/s12885-020-07092-w)

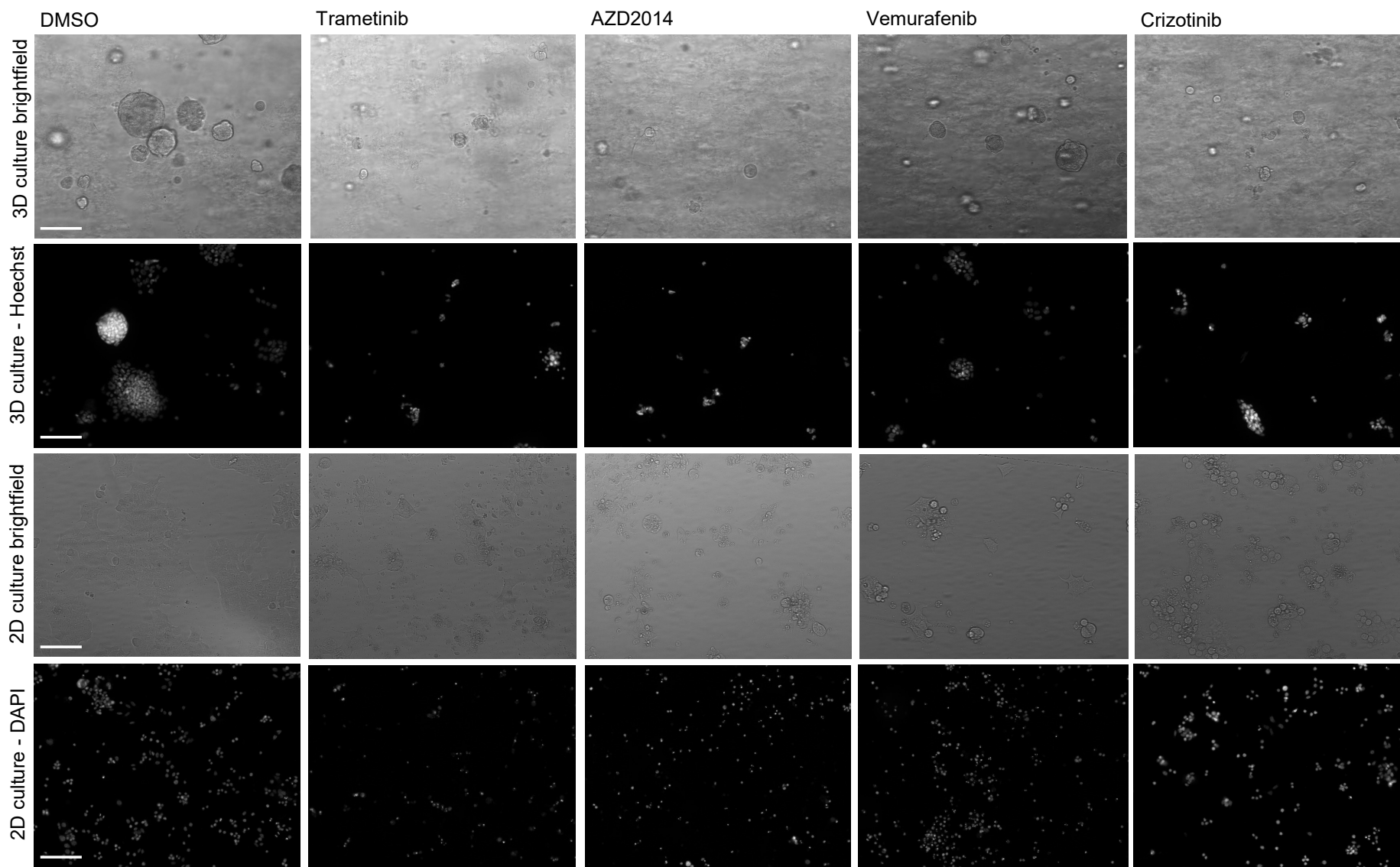

Supplement: Supplementary file 1 — Additional file 1: Figure S1. Microscopic imaging of 2D and 3D urachal cancer cell cultures. Example transmitted light microscopy images of the phenotypes for the cytotoxic drugs at 1250 nM concentration identified as potential therapeutics for urachal cancer cells based on the 2D and 3D ex vivo drug screening. Both brightfield microscopy and fluorescence microscopy imaging with DNA counterstaining with Hoechst (3D cultures) and DAPI (2D cultures) was performed with a 10× objective on an Olympus scan^R high content imager, bars 100 μm. [file 12885_2020_7092_MOESM1_ESM.pdf]

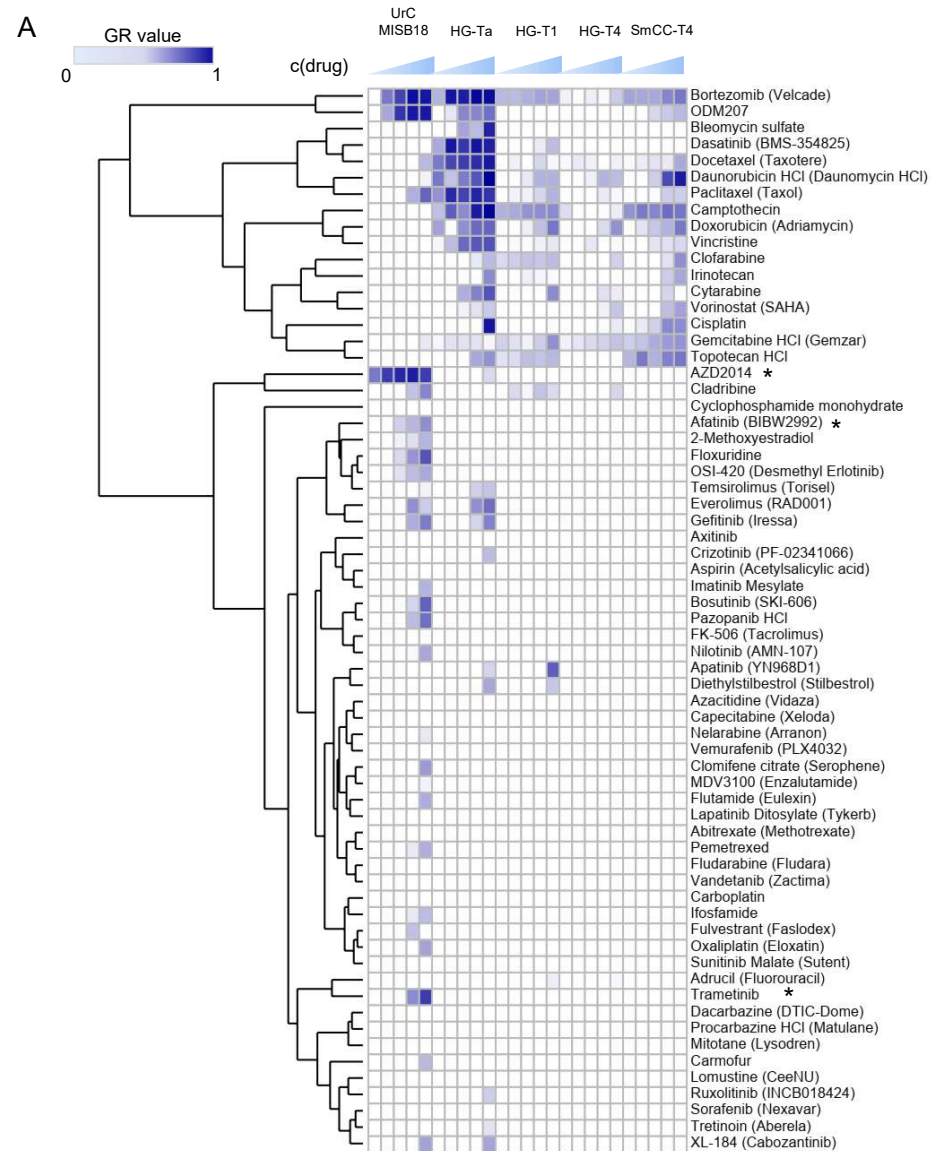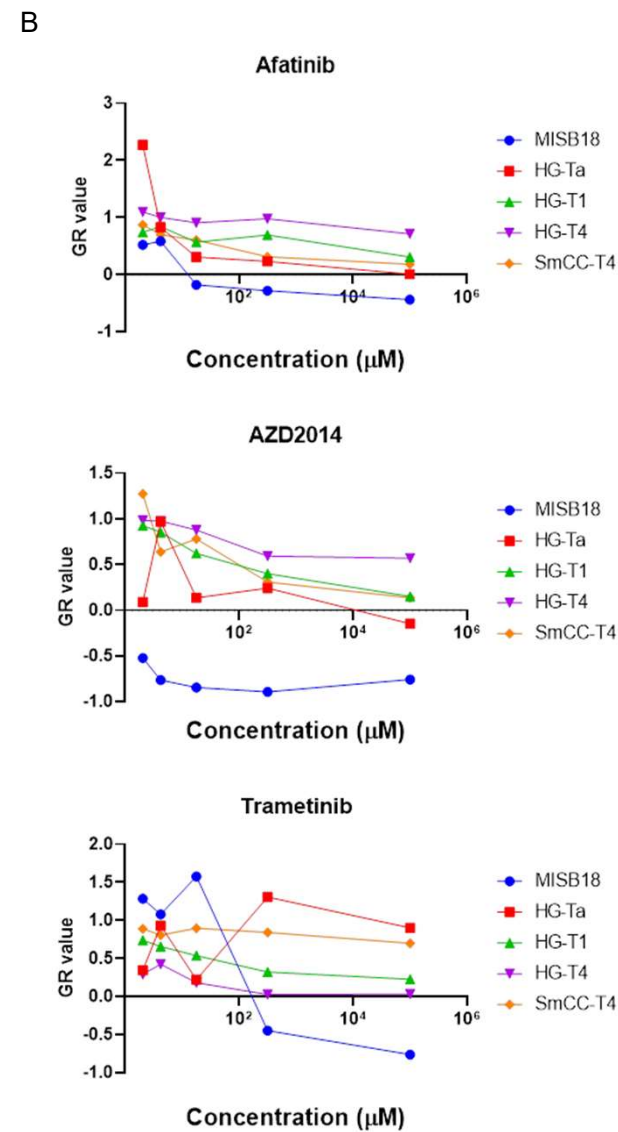

Supplement: Supplementary file 2 — Additional file 2: Figure S2. Comparison of the drug primary urachal cancer cell culture to patient derived bladder cancer cell cultures. A) Heatmap visualization of the dose response of the urachal cancer cells MISB18 with four cell cultures established from patients samples of different bladder cancer types. GR values of < 0 shown in blue. B) GR metrics describing the sensitivity of the cells to three drugs; afatinib, AZD2014 and trametinib displaying strongest selective cytotoxic effects on the urachal cancer cells in comparison to the bladder cancer cell cultures (data from image-based screening assays). Data available at Mendeley Data; DOI: https://doi.org/10.17632/kc7wmn3rcs.2. [file 12885_2020_7092_MOESM2_ESM.pdf]
